# Monte Carlo Micro-Stress Field Simulations in Flax/E-Glass Composite Laminae with Non-Circular Flax Fibres

**DOI:** 10.3390/polym17050674

**Published:** 2025-03-02

**Authors:** Nenglong Yang, Zhenmin Zou, Constantinos Soutis, Prasad Potluri, Kali Babu Katnam

**Affiliations:** 1 Department of Mechanical and Aerospace Engineering, University of Manchester, Manchester M13 9PL, UK; nenglong.yang@manchester.ac.uk (N.Y.); zhenmin.zou@manchester.ac.uk (Z.Z.); 2 Department of Materials, University of Manchester, Manchester M13 9PL, UK; constantinos.soutis@manchester.ac.uk (C.S.); prasad.potluri@manchester.ac.uk (P.P.); 3 Northwest Composites Centre, University of Manchester, Manchester M13 9PL, UK

**Keywords:** natural fibre hybrid composites, computational micromechanics, natural fibre shape, mechanical behaviour, random microstructure distribution, stochastic homogenisation

## Abstract

This study explores the mechanical behaviour of intra-laminar hybrid flax/E-glass composites, focusing on the role of micro-scale irregularities in flax fibres. By employing computational micromechanics and Monte Carlo simulations, it analyses the influence of flax fibre geometry and elastic properties on the performance of hybrid and non-hybrid composites. A Non-Circular Fibre Distribution (NCFD) algorithm is introduced to generate microstructures with randomly distributed non-circular flax and circular E-glass fibres, which are then modelled using a 3D *representative volume element* (RVE) model developed in Python 2.7 and implemented with *Abaqus/Standard*. The RVE dimensions were specified as ten times the mean characteristic length of flax fibres (580 μm) for the width and length, while the thickness was defined as one-tenth the radius of the E-glass fibre. Results show that Monte Carlo simulations accurately estimate the effect of fibre variabilities on homogenised elastic constants when compared to measured values and Halpin-Tsai predictions, and they effectively evaluate the fibre/matrix interfacial stresses and von Mises matrix stresses. While these variabilities minimally affect the homogenised properties, they increase the presence of highly stressed regions, especially at the interface and matrix of flax/epoxy composites. Additionally, intra-laminar hybridisation further increases local stress in these critical areas. These findings improve our understanding of the relationship between the natural fibre shape and mechanical performance in flax/E-glass composites, providing valuable insights for designing and optimising advanced composite materials to avoid or delay damage, such as matrix cracking and splitting, under higher applied loads.

## 1. Introduction

Natural fibres have gained increasing attention in recent years as reinforcement materials in composite applications across industries such as sports, automotive, pharmaceuticals, and construction. This is largely attributed to their energy-efficient fabrication, affordability, and lightweight properties, which align with efforts to reduce carbon footprints [1]. However, using natural fibres in composites presents challenges, such as inadequate interfacial adhesion, moisture resistance, and thermal stability. These challenges are further compounded by the inherent variability of natural fibres, which are characterised by variations in cross-sectional shapes and lengths, both within and between fibre batches [2]. Unlike synthetic fibres, which exhibit uniform circular cross-sections, natural fibres are derived from plant sources, resulting in a range of properties because of their organic origins.

Experimental studies on natural fibre-reinforced polymer composites (NFRCs) often focus on the effects of variations in fibre cross-sectional shapes and their mechanical properties [3,4,5,6]. However, these experiments face challenges in material selection, sample preparation, and testing procedures. To address these challenges, micro-mechanical models have emerged as valuable tools for predicting the mechanical behaviour and identifying the failure mechanisms of composites. While many existing micro-mechanical models for synthetic fibre reinforced polymer composites assume uniform circular sections [7], this assumption does not hold for natural fibres due to their irregular shapes. To overcome this issue, computational micromechanics approaches have been adopted in several studies [8,9,10,11,12,13,14,15], employing a *representative volume element* (RVE) model [16] along with *periodic boundary conditions* (PBCs) [17] to determine the homogenised properties of composites with non-circular fibres. Nevertheless, concerns persist due to the variability in the ability of well-established micro-mechanical models to accurately represent the complex geometries and behaviours of irregularly shaped fibres in composites [18]. Specifically, questions arise regarding whether variations in the cross-sectional area along a single natural fibre significantly affect its modulus. To address this, a study by Thomason et al. [7] revealed that experimental variability in the measured fibre modulus values primarily arises from factors other than variations in the cross-sectional area along a single natural fibre. Therefore, adopting a consistent cross-sectional area for the fibres along their length is a reasonable assumption for micro-mechanical modelling. Moreover, while the shape factor, defined by the cross-sectional perimeter of the fibres, contributes to the multi-physical properties of fibre-reinforced composites, it has a limited effect on their longitudinal properties [19].

Various irregular fibre shapes have been explored, including triangular [3,4,8,9], kidney-type [5], C-type and hollow type [6], lobular, polygonal and elliptical [10], Reuleaux and star-type [11], gear [12] and oval [15] shapes. For instance, triangular glass fibre-reinforced polymer composites have demonstrated a 20% increase in tensile strength and a 40% increase in compressive strength [3]. Similarly, triangular carbon fibre-reinforced composites exhibit improved transverse stiffness and strength [8,9], as their triangular cross-sectional shape effectively restrains matrix deformation. Additionally, triangular carbon fibre-reinforced polymer composites showed a 19.8% increase in flexural strength, due to the larger contact area, allowing more efficient load transfer and a stronger binding force at the interface [4]. Experimental studies have revealed that kidney-type fibre-reinforced composites outperform circular fibre-reinforced composites under interfacial shear and interlaminar shear loads [5]. Moreover, C-type and hollow-type fibre-reinforced polymer composites show superior thermal conductivity and mechanical performance [6]. These improvements arise from stronger interfacial bonding and a larger contact surface between the matrix and fibres, facilitating improved stress transfer within the material [5,6]. Lobular fibres demonstrate higher tensile residual stresses and compressive strength, primarily due to increased interfacial stresses [10]. Star-shaped fibres significantly improve the transverse and shear stiffness while reducing the damping [11]. In contrast, elliptical and Reuleaux fibres moderately improve damping at the expense of stiffness. Although gear-shaped fibre-reinforced composites exhibit superior transverse stiffness and strength compared to circular counterparts, their complex manufacturing process significantly increases costs. Interestingly, reducing the number of teeth increases the bonding surface area, which strengthens the fibre/matrix adhesion and mitigates matrix cracking and interfacial debonding [12]. However, oval-shaped fibres show reduced mechanical performance, with their strength and toughness compromised under transverse compression due to misalignments, although these misalignments have a minimal effect under tensile transverse loading [15]. Additionally, when fibres touch and interlock, a crack in one fibre easily transfers to neighbouring fibres, leading to a more brittle fracture and premature failures.

The effectiveness of load transfer in NFRCs is heavily dependent on the bonding and adhesion established at the interface between the natural fibres and surrounding matrix. Micro-mechanical models typically consider two types of interfaces: one with zero thickness and the other with finite thickness. The stiffness, strength, and toughness of these interfaces significantly influence the overall mechanical behaviour of the composites. Interface degradation leads to a decrease in adhesion and bond strength in composites. Prior studies [20,21,22] have explored how the microstructure and fibre/matrix interface affect the mechanical properties of carbon fibre-reinforced composites under transverse tension. They identified key factors, including the fibre alignment angle and minimum inter-fibre distance, which affect the interfacial stresses and damage mechanisms. Specifically, Hojo et al. [22] found that tensile interfacial normal stresses occur in regions where inter-fibre distance is minimal and alignment angles are minimal relative to the loading direction. Their study revealed that interfacial debonding plays a key role in damage initiation, whereas damage propagation is governed by shear-band formation and subsequent matrix cracking. These dominant damage modes are consistently recognised as key factors affecting composite strength [10,23,24,25,26].

The inherent variability in natural fibres results in inconsistent structural performance within NFRCs. To address this, synthetic fibres are incorporated into the composites to reduce the overall variation in mechanical performance, resulting in what is known as a natural/synthetic fibre-reinforced hybrid composite (NFHC). These NFHCs are manufactured by combining natural and synthetic fibres either between different layers (inter-laminar) or within multiple layers (intra-laminar) [27]. Intra-laminar hybridisation of NFHCs has been shown to enhance both flexural and inter-laminar shear strength [28], along with improving overall damage tolerance [29,30,31,32]. While micro-mechanical modelling is standard for predicting the mechanical properties of synthetic fibre-reinforced polymer composites, the influence of natural fibre cross-sectional irregularities on NFHCs has received little attention. Existing models assume that fibres are circular, neglecting the variability in shape within fibre batches and its implications for accurately predicting the mechanical response of composites with non-circular fibres.

This study aims to fill this gap by analysing, through computational methods, how natural fibre shapes influence the mechanical performance of NFHCs, as well as their effect on fibre/matrix interfacial stress distribution and von Mises matrix stress. Employing a Monte Carlo simulation (MCS) approach, the fibre-level variabilities inherent in the composites are considered. This method, commonly used to evaluate variability in input parameters through multiple simulations based on sampled distributions [33,34,35,36,37,38,39,40,41,42], has not previously been applied to examine the influence of irregular natural fibre shape in NFHCs. In this study, a stochastic homogenisation approach incorporating MCS is employed, with a micro-mechanical RVE model implemented in Python and Abaqus/Standard [43]. A Non-Circular Fibre Distribution (NCFD) algorithm is introduced to randomly create microstructures that consist of circular E-glass fibres and irregular flax fibres, with irregular flax fibre sizes following a normal distribution. An uncertainty analysis is conducted to explore how variations in the flax fibre shape and elastic properties influence the homogenised elastic properties, interfacial stresses, and von Mises matrix stress fields in NFHCs.

## 2. Stochastic Homogenisation via Monte Carlo Simulation

A micro-mechanical RVE model was used to analyse the mechanical behaviours of unidirectional hybrid flax/E-glass and non-hybrid flax composite laminae. This model incorporated a random arrangement of circular E-glass and irregularly shaped flax fibres. Homogenised elastic constants and micro-scale stress fields were determined under three axial tensile and three shear loadings. Variability in the flax shape and elasticity was systematically addressed within the simulations by employing normal distributions derived from experimental data on flax fibre properties as input parameters.

### 2.1. Hybrid and Non-Hybrid Composite Microstructures

**Figure 1:**
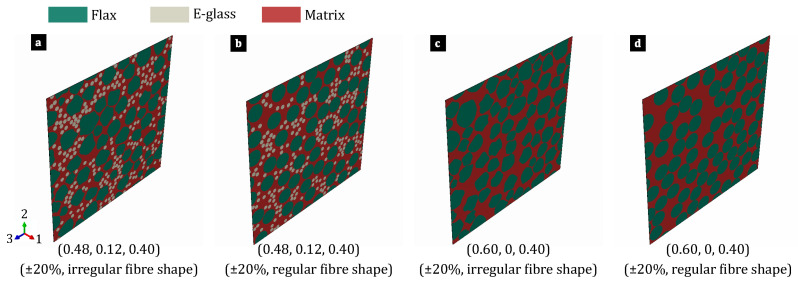
Representative microstructures of hybrid and non-hybrid composites created using the NCFD algorithm, showing the effects of varying flax fibre shapes and the specified fibre volume fractions for flax, E-glass, and matrix ($V_{fF},{V_{fE},V}_{m}$) as follows: (**a**) (0.48, 0.12, 0.40), (**b**) (0.48, 0.12, 0.40), (**c**) (0.60, 0, 0.40), (**d**) (0.60, 0, 0.40).

As shown in Figure 1, the 3D RVE models were developed to analyse the microstructure of the hybrid flax/E-glass and non-hybrid flax composite laminae. These models incorporated variations in the shapes of flax fibres. In the RVEs, it was assumed that the parallel continuous fibres were randomly distributed within the matrix, with their cross-sectional shape remaining constant throughout their length. However, in reality, flax fibres usually vary from 10 to 65 mm in length [44]. The constituents were considered homogeneous and void-free. E-glass fibres are modelled as having a circular cross-section, while flax fibres may have either circular or irregular cross-sections. It is also assumed that the bond between both types of fibres and the matrix is perfect, preventing any interfacial separation under any loading condition. To better understand the mechanical behaviour of hybrid composites, this study examines the variability in both the flax fibre geometry and elastic properties. In contrast, the diameter and elastic properties of the E-glass fibres and the matrix properties are treated as constants. A probability density function (PDF) based on a normal distribution is used to capture this variability in the material properties. In Equation (1), the PDF represents the material properties as random variables (x), parameterised by their mean (μ) and the standard deviation (σ).

(1) $$f(x)=\frac{1}{\sigma \sqrt{2\pi }}e^{-\frac{1}{2}{\left(\frac{x-\mu }{\sigma }\right)}^{2}}$$

Flax fibre characteristics are inherently variable because of the natural variability introduced by factors like plant growth stages, soil conditions, and seasonal variation, making their measurement challenging. This study proposes a method to incorporate this variability by employing normal distributions, which provides a framework for representing the stochastic nature of flax fibre characteristics. Table 1 presents the properties of flax fibres compiled from multiple sources [27,45,46,47,48,49]. The diameter of flax fibres exhibits significant variation, ranging from 5 μm [45] to 185 μm [47], while the tensile modulus also varies substantially, from 27.5 GPa [46] to 90 GPa [27]. To accommodate this variability, both the elastic properties and the mean diameter (d) are assumed to follow normal probability distributions. Each distribution is assigned a coefficient of variation (COV) of 20%, calculated by dividing the standard deviation by the mean, as shown in Equation (2). The mean characteristic length of flax fibres is 58 μm, with upper and lower bounds of 23.2 μm and 92.8 μm, based on the average of measurements obtained in references [27,45,46,47,48,49]. To address the variability in the measurements, a μ±3σ range (i.e., 34.8 μm) was applied to the measurements.

(2) $$COV=\frac{\sigma }{\mu }\times 100\%$$

To analyse composites at the microscopic level, it is essential to generate an RVE that adequately represents the material on a large scale while being appropriately sized. In determining the RVE dimensions, the mean diameter of the irregular flax fibre was considered the representative length for the microstructure. For the flax/E-glass composites, the width and length of the RVE were set to approximately 580 μm, which is ten times the average characteristic length of the irregular flax fibres. With no stress gradients along the fibre direction, only one thin layer of elements is required [50]. Furthermore, the chosen RVE size meets the criteria set by Trias et al. [51] for homogenisation and ensures the inclusion of a sufficient number of flax fibres within the domain, thereby guaranteeing an accurate representation of the studied material.

The NCFD algorithm was developed to create the microstructures of the unidirectional hybrid flax/E-glass and non-hybrid flax composite laminae while avoiding the overlap of irregularly shaped flax fibres for FEA analysis. Both deterministic and stochastic micro-mechanical models were employed for the analysis. In the deterministic model, the flax fibre cross-sectional shape and elastic properties were assumed to be constant, whereas the stochastic model accounted for variations in both. All simulations maintained a fibre volume fraction of 0.6, with an error margin of ±0.02% to ensure minimal variability across microstructures. Specifically, the constituent volume fractions were as follows: flax fibres $\left(V_{fF}\right)=0.48$, E-glass fibres $\left(V_{fE}\right)=0.12$ and matrix $\left(V_{m}\right)=0.40$. The NCFD algorithm, implemented in MATLAB R2023b, was used to generate microstructures containing fibres with constant circular or irregular cross-sectional shapes. The algorithm initially generated microstructures with circular fibres and then modified them to achieve irregular cross-sectional shapes for the flax fibres.

During the preprocessing stage, a shape library was established by incorporating concepts from prior works [13,14,15]. This library contains geometric details describing the cross-sectional shapes of circular E-glass fibres and irregular flax fibres. Circular E-glass fibres were characterised by their centre coordinates and diameter, whereas irregular flax fibres were defined by a contour formed by connecting 50 points using cubic spline interpolation. Initially, 12 points were evenly distributed along the circumference of a circular fibre, with fibre diameters following a normal distribution. These points were then randomly adjusted within intervals of ±20% of the mean radius of the irregularly shaped flax fibres. The adjusted points were connected using a closed parametric cubic spline curve with the MATLAB function “cscvn”, and evaluated with 1500 evenly spaced points using the MATLAB function “ppval”. Parameters corresponding to 50 evenly spaced arc length values were determined using the MATLAB function “pdearcl”, and the curve was re-evaluated at these points with “ppval”. The area and volume fractions of the irregular flax fibres were calculated based on 50 points. While determining the area of circular E-glass fibres is straightforward, the process differs for irregular flax fibres. The area of the irregularly shaped flax fibres was computed using the MATLAB function “integral”, employing global adaptive quadrature and default error tolerances. A random shape library was iteratively produced until the volume fraction of each constituent fell within a strict tolerance range of ±0.2%. The fibres from the shape library were ordered by size and added sequentially into the domain at random locations, with each fibre placed near the previously placed fibres in the RVE domain. Initially, a check ensured that no fibre edges were too close to the RVE domain edge, with an acceptable position defined as having a distance from the edge exceeding 0.3 μm. This threshold value was selected to generate a finite element mesh with acceptable quality. Subsequently, overlapping was assessed based on the fibre types. For two circular fibres, their centres needed to be more than the sum of their radii plus an additional 0.3 μm apart to avoid overlap. For circular and irregular fibres, all 50 points defining the irregular fibre had to be located at a distance greater than the radius of the circular fibre plus twice the 0.3 μm tolerance, accounting for its spline nature. Neglecting this tolerance may lead to an undesired fibre overlap. Overlapping checks between two irregular fibres involved a two-step process: first, the MATLAB function “polyshape” converted the fibres into polygon-like shapes, and then the MATLAB function “overlaps” detected any overlap between them. Fibres were accepted if they did not overlap with existing fibres; otherwise, they were rejected.

Moreover, a periodicity check ensures the periodic nature of the microstructure within the RVE domain. When a fibre intersects the edge of the domain, its remaining portion outside the domain is replicated on the opposite edge, subject to prior checks. If the checks fail, alternative positions within the RVE are sought. For each fibre in the RVE, the NCFD algorithm attempts to find a new position by making 360 attempts around the existing fibre to fill all available space with new fibres. To enhance efficiency, the RVE is partitioned into smaller second-level sub-squares, each with dimensions equal to the mean characteristic length of the irregularly shaped fibres. When positioning a new fibre, the NCFD algorithm identifies the sub-square containing its centre and conducts overlapping checks within the adjacent eight sub-squares. The iteration continues until all attempts are made, or the desired fibre volume fraction is achieved. After completing the NCFD algorithm, the centre coordinates of the circular E-glass fibres and 50 points for each irregular flax fibre are exported to a text file, readable by *Abaqus/Standard* for microstructure generation.

### 2.2. Micro-Mechanical Stochastic RVE FE Model

The RVE was discretised using a combination of tetrahedral (C3D6) and hexahedral (C3D8R) elements with a uniform mesh distribution applied across the domain. This meshing approach ensured consistent element quality throughout the RVE while accurately capturing the complex geometries of both the circular E-glass and irregular flax fibres. To determine the optimal average element size, a mesh independent analysis was conducted. An average size equal to 1/10 of the E-glass fibre’s radius (approximately 0.75 μm for the hybrid composites), was used for meshing with one layer of elements in the fibre direction. Although this mesh size is smaller than the previously recommended optimal values [41,52,53], the chosen size achieved convergence in the elastic constants of the composites. It also ensured both mesh independence in the simulation results and an efficient computational performance while maintaining the statistical independence of the results.

To characterise the microstructure of the composite laminae, an RVE is employed. Given that this study focuses on uncertainty analysis at the micro-scale, only the key details of the established RVE model are presented here. For a more comprehensive discussion of the model formulation and implementation, the reader is referred to [54]. This RVE can be infinitely translated along two perpendicular axes while maintaining the periodicity of the fibre positions. This periodicity is maintained by applying displacement-based PBCs between the opposing faces of the RVE, ensuring continuity in both the micro-scale stress/strain and displacement fields. The implementation details of these PBCs in *Abaqus/Standard* are provided in [50]. The RVE was subjected to six load cases using six reference points: (a) longitudinal tension (${\hat{\sigma }}_{11}=1\;MPa),\;$(b) transverse tensions (${\hat{\sigma }}_{22}=1\;MPa$ or ${\hat{\sigma }}_{33}=1\;MPa)$, (c) in-plane shears (${\hat{\sigma }}_{12}=1\;MPa$ or ${\hat{\sigma }}_{13}=1\;MPa)$, and (d) out-of-plane shear (${\hat{\sigma }}_{23}=1\;MPa$). Each macroscopic stress component (${\hat{\sigma }}_{11},{\hat{\sigma }}_{22},{\hat{\sigma }}_{33},{\hat{\sigma }}_{12},{\hat{\sigma }}_{13}$ and ${\hat{\sigma }}_{23}$) is directly correlated with the generalised nodal forces (${\hat{F}}_{11},{\hat{F}}_{22},{\hat{F}}_{33},{\hat{F}}_{12},{\hat{F}}_{13}\;and\;{\hat{F}}_{23}$) at each reference point. The relationship between these forces, the macroscopic stresses, and the RVE volume ($\hat{\Omega }$) is governed by the energy equivalence principle, as shown in Equation (3) [50]. The subscript “1” refers the longitudinal fibre direction, while “2” and “3” correspond to the transverse directions.

(3) $${\hat{F}}_{ij}={\hat{\sigma }}_{ij}\hat{\Omega }\;\;\left(i,j=1,\;2,\;3\right)$$

Similarly, the six macroscopic strain components $\left({\hat{\varepsilon }}_{11},{\hat{\varepsilon }}_{22},{\hat{\varepsilon }}_{33},{\hat{\varepsilon }}_{12},{\hat{\varepsilon }}_{13},{\hat{\varepsilon }}_{23}\right)$ at every reference point are directly linked to the generalised nodal displacements. Moreover, the relationship between these forces and displacements is established through the PBCs. By applying six independent loadings, each representing generalised nodal forces at their corresponding reference points, the corresponding nodal displacements can be obtained. These nodal displacements can be used to obtain the compliance tensor, which, in turn, predicts the homogenised elastic constants for the laminae. In the case of longitudinal tension, the sole applied loading is the generalised nodal force ${\hat{F}}_{11}$, with its value set to $\hat{\Omega }$. This loading case simulates a uniaxial tensile load, resulting in a macroscopic stress component ${\hat{\sigma }}_{11}=1$, as expressed in Equation (3). At the reference point, the generalised nodal displacements represent the three macroscopic strain components (${\hat{\varepsilon }}_{11},{\hat{\varepsilon }}_{22},{\hat{\varepsilon }}_{33}$). The homogenised properties (${\hat{E}}_{11}$, ${\hat{\nu }}_{12}$, ${\hat{\nu }}_{13}$) can then be predicted using Equations (4)–(6) [50]. Using a similar approach, the remaining homogenised elastic constants are predicted for other loading cases, resulting in a complete set of homogenised elastic constants (${\hat{E}}_{11}$, ${\hat{E}}_{22}$, ${\hat{E}}_{33},\;{\hat{G}}_{12}$, ${\hat{G}}_{13}$, ${\hat{G}}_{23},\;{\hat{\nu }}_{12}$, ${\hat{\nu }}_{13}$, ${\hat{\nu }}_{23}$) for the composites.

(4) $${\hat{E}}_{11}=\frac{{\hat{\sigma }}_{11}}{{\hat{\varepsilon }}_{11}}=\frac{1}{{\hat{\varepsilon }}_{11}}$$

(5) $${\hat{\nu }}_{12}=-\frac{{\hat{\varepsilon }}_{22}}{{\hat{\varepsilon }}_{11}}$$

(6) $${\hat{\nu }}_{13}=-\frac{{\hat{\varepsilon }}_{33}}{{\hat{\varepsilon }}_{11}}\;$$

The properties of the input material properties, fibre diameters, and their distributions are summarised in Table 2. Isotropy was assumed for the matrix and E-glass fibres, with flax fibres treated as transversely isotropic. The nominal properties of flax fibres were obtained solely from reference [55], with tensile modulus values obtained from various studies. Numerical stability in generating elastic properties aligned with a normal distribution was achieved by enforcing the constraints described in Equations (7)–(11) [56].

(7) $$E_{1},E_{2},E_{3},G_{12},G_{13},G_{23}>0$$

(8) $$\left|\nu_{12}\right|<{\left(E_{1}/E_{2}\right)}^{1/2}$$

(9) $$\left|\nu_{13}\right|<{\left(E_{1}/E_{3}\right)}^{1/2}$$

(10) $$\left|\nu_{23}\right|<{\left(E_{2}/E_{3}\right)}^{1/2}$$

(11) $$1-\nu_{12}\nu_{21}-\nu_{23}\nu_{32}-\nu_{31}\nu_{13}-2\nu_{21}\nu_{32}\nu_{13}>0$$

The interfacial normal/shear stresses within the interface regions and von Mises matrix stress are computed at each integration point of the elements. The normal stress ($\sigma_{n}$) is perpendicular to the interface, whereas the shear stress ($\tau_{nt}$) follows the circumference of the fibre in a tangential direction. Both are expressed in local material orientations. This orientation is established for all interface regions using discrete orientation fields, each defined by a normal axis and a primary axis. The surface of the fibre/matrix interface defines the normal axis, which follows the fibre direction, while the primary axis follows the circumference of the fibres.

### 2.3. Monte Carlo Uncertainty Analysis

The Monte Carlo Simulation (MCS) framework provides a robust method for predicting the homogenised elastic constants of composite laminae. This method involves a series of simulations aimed at gradually converging the sample to accurately represent the overall population [59]. More simulations improve the accuracy but also increase the computational time. To optimise the uncertainty analysis, the minimum number of samples (n) for a specific confidence level while maintaining a particular relative error (δ) can be calculated using Equation (12) [59]. To achieve a 95% confidence level with a relative error (δ) of 0.05 in this study, a minimum of 62 simulations are required for flax fibres modelled under a normal distribution (COV=20%). The Z-score $z_{c}=1.96$ corresponds to the 95% confidence interval, defining a range of ±1.96 times the standard deviation from the mean.

(12) $$n={\left[\frac{z_{c}COV}{\delta }\right]}^{2}$$

The microstructures were individually generated for each RVE. Subsequently, meshes were created, PBCs were applied, and loadings were imposed, all automated through Python scripts. Table 3 summarises the eight MCS cases that were conducted, comprising 520 simulations. These cases explore the effect of variations in the cross-sectional shapes and elastic properties of fibres on the mechanical properties, interfacial stresses, and von Mises stress distribution in the composites while considering the inherent uncertainty in fibre positions. Each MCS case comprises 65 simulations: cases 1–4 focused on hybrid flax/E-glass composites, and cases 5–8 focused on non-hybrid flax composites. For hybrid flax/E-glass, MCS case 1 served as a baseline, examining RVEs containing flax fibres with a ±20% variation in both shape and elastic properties. Conversely, MCS case 2 examined the RVEs containing flax fibres with no variation in shape or elastic properties. MCS cases 3 and 4 individually examined RVEs containing flax fibres with a ±20% variation in shape and elastic properties. Similarly, MCS cases 5–8 were conducted for flax composites, examining the role of fibre hybridisation in this context.

## 3. Influence of Fibre-Level Variabilities

MCS-based stochastic homogenisation was first employed to investigate non-hybrid flax composites. Table 4 summarises the mesoscopic elastic constants of the flax/PP/MAPP epoxy [60] and E-glass/MY750 epoxy [61] composites using the results from the RVE model, experimental data, and Halpin-Tsai model [62]. For flax composites, the RVE model predicts the longitudinal modulus (${\hat{E}}_{11}$) at 31.68 GPa, closely matching the experimental measurement (34.40 GPa) and the Halpin-Tsai prediction (31.84 GPa). While experimental data for other properties are unavailable, the RVE predictions for E-glass/epoxy composites demonstrate a strong agreement with both analytical and experimental results.

In particular, the ${\hat{E}}_{11}$ is predicted to be 45.56 GPa, which closely matches the Halpin-Tsai prediction and the experimental measurement. Additionally, the ${\hat{E}}_{22}$, ${\hat{G}}_{12}$ and ${\hat{G}}_{23}$ predicted by the RVE model align well with the experimental measurements and analytical model. The close agreement between the experimental and analytical results and RVE predictions validate the RVE model employed in this study, despite the limited experimental data for the flax composites. Furthermore, with negligible standard deviation in the homogenised elastic constants and reduced error compared to the Halpin-Tsai prediction, the chosen RVE and element sizes are confirmed to be well-suited.

### 3.1. Analysis of Fibre-Level Variabilities on Homogenised Elastic Constants

**Figure 2:**
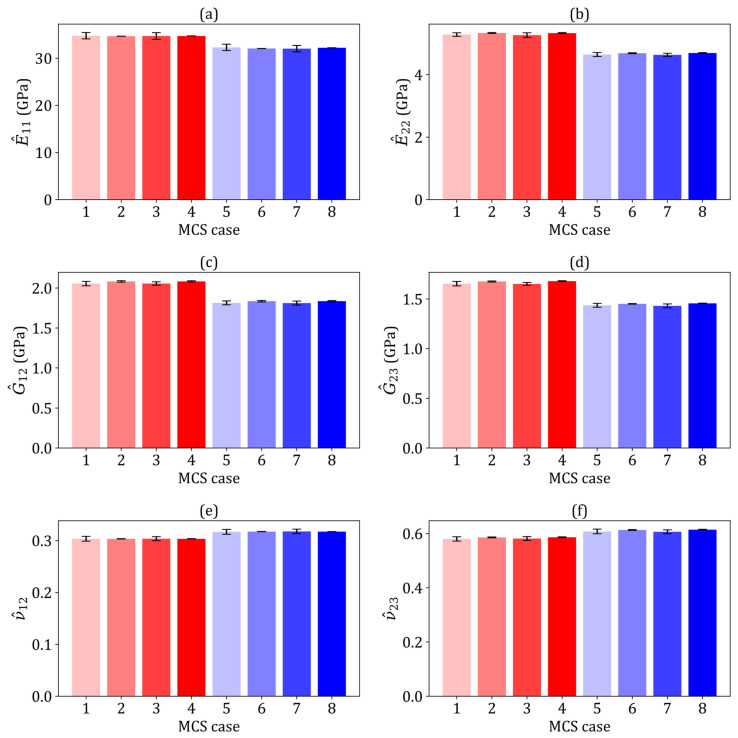
The mean homogenised elastic constants computed from eight Monte Carlo simulation cases, with colour-coded bars transitioning from light to dark red for MCS cases 1–4 (flax/E-glass composites) and light to dark blue for MCS cases 5–8 (flax composites): (**a**) ${\hat{E}}_{11}$ (GPa), (**b**) ${\hat{E}}_{22}$ (GPa), (**c**) ${\hat{G}}_{12}$ (GPa), (**d**) ${\hat{G}}_{23}$ (GPa), (**e**) ${\hat{\nu }}_{12}$ and (**f**) ${\hat{\nu }}_{23}$.

The robustness of the MCS-based stochastic model was confirmed by assessing the convergence of the mean values (μ) and coefficient of variation (COV) for the homogenised elastic constants of composites in each MCS case. After 40 simulations, the μ and COVs of all homogenised elastic constants converged, with a relative difference of 3%. Thus, 65 simulations per MCS case were considered adequate for this study. Figure 2 summarises the homogenised elastic constants of the composites across the MCS cases. The homogenised properties of the flax composites, regardless of variations in fibre shape or elastic properties, were closely aligned with those without such variations (MCS cases 5–8). These mean values are consistent with the analytical prediction (see Table 4) and the findings of Higuchi et al. [8], suggesting minimal variation in the mechanical properties across different fibre shapes in random distributions. Moreover, for the hybrid composites (MCS cases 1–4), the mean homogenised elastic constants were consistent. The overall uncertainty in these constants was minimally influenced by the elastic properties, position, and shape of the fibres. While differences in individual fibre stiffness influenced the localised RVE stiffness, their effects were balanced within each sufficiently large RVE. Attempts to increase the RVE size to assess its effect on the mean homogenised properties showed minimal changes, although there was a reduction in COVs, further supporting that the RVE sizes used in this study were appropriate.

A normal distribution with a COV of 5% was commonly assumed in studies addressing the elastic properties of carbon fibre [34,36,63,64]. However, the COVs reported for the homogenised elastic constants of carbon/epoxy laminae varied: 4.5% [34], 6.4% [36], 7.2% [63] and 5.6% [64]. These average COVs were either similar to or greater than the initially assumed input COVs. However, in contrast to these findings, the present study reveals a significant difference: all COVs for the homogenised elastic constants were approximately 10 times smaller than the initially assumed input COVs. This discrepancy arises from the distinct modelling approaches. Stochastic RVE models simulate flax fibres with random distributions, each flax fibre having unique elastic properties and cross-sectional shapes that conform to normal distributions. In contrast, a square RVE model containing a single fibre does not account for these inherent variations [64]. Since flax fibres exhibit normally distributed elastic properties, increasing the number of fibres in the RVE model reduces the COVs for the homogenised elastic constants relative to the input COVs. Consequently, this decrease in COVs reduces the variability within the composite laminae.

### 3.2. Analysis of Fibre-Level Variabilities on Micro-Stress Fields

**Figure 3:**
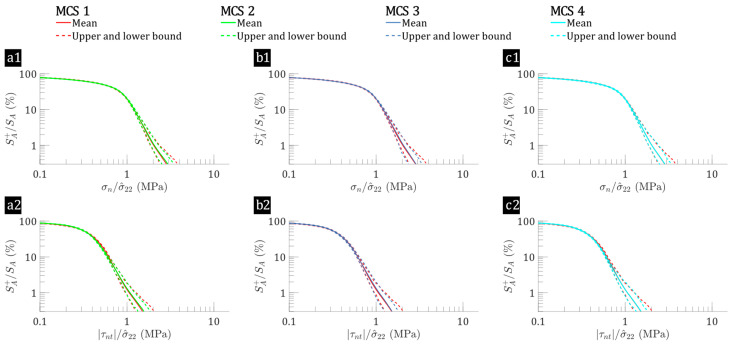
Interface reversed cumulative surface percentages as functions of interfacial stresses: (**a1**,**b1**,**c1**) $\sigma_{n}$, (**a2**,**b2**,**c2**) $\left|\tau_{nt}\right|$, for flax/E-glass composites (MCS cases 1–4) subjected to transverse tension (${\hat{\sigma }}_{22}=1$ MPa), where the leftmost point represents the percentage of interface regions experiencing normalised interfacial stresses greater than 0.1, progressively decreasing rightward to illustrate the proportion subjected to increasing stress, with MCS 1 (baseline) shown by the red line, which may be partially obscured when comparing data from MCS 2, MCS 3, and MCS 4 in each subplot.

**Figure 4:**
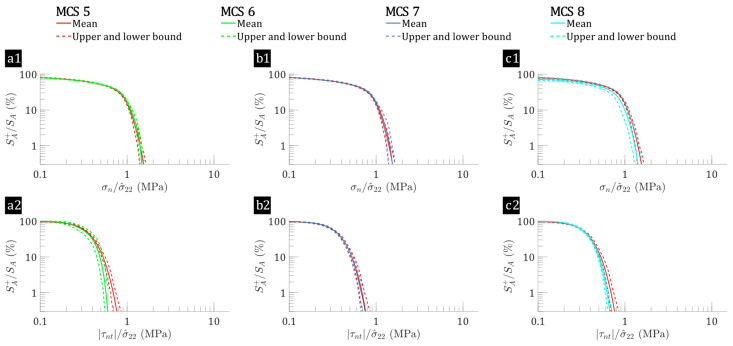
Interface reversed cumulative surface percentages as functions of interfacial stresses: (**a1**,**b1**,**c1**) $\sigma_{n}$, (**a2**,**b2**,**c2**) $\left|\tau_{nt}\right|$, for flax composites (MCS cases 5–8) subjected to transverse tension (${\hat{\sigma }}_{22}=1\;MPa)$, where the leftmost point represents the percentage of interface regions experiencing normalised interfacial stresses greater than 0.1, progressively decreasing rightward to illustrate the proportion subjected to increasing stress, with MCS 5 (baseline) shown by the red line, which may be partially obscured when comparing data from MCS 6, MCS 7, and MCS 8 in each subplot.

**Figure 5:**
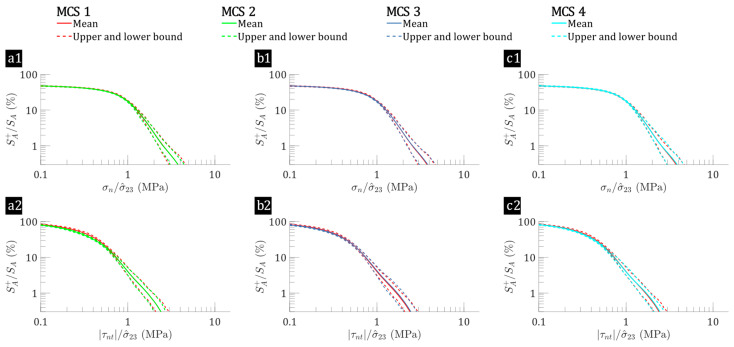
Interface reversed cumulative surface percentages as functions of interfacial stresses: (**a1**,**b1**,**c1**) $\sigma_{n}$, (**a2**,**b2**,**c2**) $\left|\tau_{nt}\right|$, for MCS cases 1–4 (flax/E-glass composites) subjected to out-of-plane shear (${\hat{\sigma }}_{23}=1\;MPa)$, where the leftmost point represents the percentage of interface regions experiencing normalised interfacial stresses greater than 0.1, progressively decreasing rightward to illustrate the proportion subjected to increasing stress, with MCS 1 (baseline) shown by the red line, which may be partially obscured when comparing data from MCS 2, MCS 3, and MCS 4 in each subplot.

**Figure 6:**
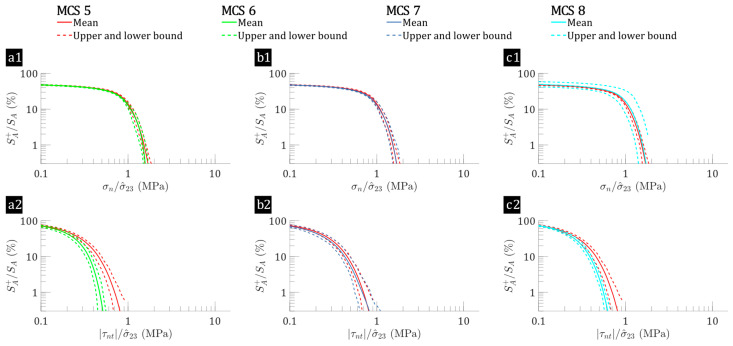
Interface reversed cumulative surface percentages as functions of interfacial stresses: (**a1**,**b1**,**c1**) $\sigma_{n}$, (**a2**,**b2**,**c2**) $\left|\tau_{nt}\right|$, for MCS cases 5–8 (flax laminae) subjected to out-of-plane shear (${\hat{\sigma }}_{23}=1\;MPa)$, where the leftmost point represents the percentage of interface regions experiencing normalised interfacial stresses greater than 0.1, progressively decreasing rightward to illustrate the proportion subjected to increasing stress, with MCS 5 (baseline) shown by the red line, which may be partially obscured when comparing data from MCS 6, MCS 7, and MCS 8 in each subplot.

The effects of fibre-level variability on interfacial stress in the interface region of hybrid flax/E-glass and non-hybrid flax composite laminae are examined. To effectively interpret the results, a reversed cumulative surface percentage approach is used to represent the relationship between the proportion of interface regions ($S_{A}^{+}/S_{A}$) and interfacial stresses, including normal stress ($\sigma_{n}$) and shear stress ($\tau_{nt}$). Two loading conditions—transverse tensile stress (${\hat{\sigma }}_{22}=1\;MPa$) and out-of-plane shear stress (${\hat{\sigma }}_{23}=1\;MPa)$—are considered, and the results are detailed in Figure 3, Figure 4, Figure 5 and Figure 6. Here, $S_{A}$ denotes the total surface area of the interface regions, while $S_{A}^{+}$ represents the subset of these regions where either tensile normal stress or non-zero interfacial shear stress (positive or negative) is present. The focus is on the interfacial normal stress and absolute values of the interfacial shear stress. This analysis covers both loading cases for all 65 microstructures per MCS case, presenting the mean interfacial stress along with its upper and lower bounds. This method clarifies the stress distribution by showing the percentage of the total interface area subjected to a specific stress level or higher. Specifically, the leftmost part of the x-axis corresponds to all interface locations (i.e., $S_{A}^{+}/S_{A}=100\%$), and as one moves to the right along the axis, the curve reveals the proportion of interface regions experiencing increasing stress levels. The axes are plotted on a logarithmic scale beginning at 0.1 and normalised with respect to the applied loadings. This approach effectively highlights high-stress regions across multiple microstructures, which is particularly valuable given the large dataset obtained from Monte Carlo simulations.

The baseline (MCS case 1) for the flax/E-glass composites considers fibre-level variabilities, while a comparison with other cases (MCS cases 2–4) reveals a consistent pattern in the low-stress region but different patterns in the highly stressed interfacial regions. As shown in Figure 3, MCS case 1 is present in all three subfigures; however, its red lines are obscured by the lines for MCS cases 2–4 due to the small differences observed in the low-stress regions. These differences become more pronounced as the curve shifts toward the higher stress region along the axis. Specifically, under transverse tension (see Figure 3(a1,b1,c1)), the mean surface percentage of the interface where $\sigma_{n}/{\hat{\sigma }}_{22}>3$ ranges from 0.08% to 0.58% in the baseline case (MCS case 1). In contrast, other cases show narrower ranges: 0.12–0.48% for MCS case 2 (fibre distribution variations), 0.08–0.47% for MCS case 3 (flax fibre elastic property variations), and 0.11–0.45% for MCS case 4 (flax fibre shape variations). These narrower ranges suggest that the variability in flax fibre shape and elastic properties leads to more interface regions experiencing high $\sigma_{n}$ values in flax/E-glass laminae under transverse tension. A similar trend is observed for $\left|\tau_{nt}\right|$, where highly stressed interfacial regions show a narrower range compared to the baseline, indicating an increase in the surface area experiencing high $\sigma_{n}$ in flax/E-glass laminae under transverse tension when considering fibre-level variabilities. Similar patterns are seen in Figure 4 for flax composite laminae, although with a higher percentage of highly stressed interfaces. Specifically, the percentage of surface regions with $\sigma_{n}/{\hat{\sigma }}_{22}>1$ ranges from 9.77% to 15.05% (MCS cases 5–8) for flax laminae, whereas for flax/E-glass laminae, it ranges from 19.74% to 20.27% (Figure 3(a1,b1,c1)), MCS cases 1–4). Although fibre-level variabilities influence stress distributions in both systems, the reversed cumulative surface curves in Figure 3 (flax/E-glass composite) and Figure 4 (flax composite) differ significantly. Flax/E-glass composites exhibit a more broader stress distribution due to the presence of stiffer E-glass fibres, increasing variability and localising stress concentrations. This highlights how intra-laminar hybridisation affects interfacial stress distribution, leading to different stress behaviours between hybrid and non-hybrid composites.

Under out-of-plane shear loading, the interfacial normal stress ($\sigma_{n}$) and shear stress ($\tau_{nt}$) distributions for the flax/E-glass composites (Figure 5) remains consistent across MCS cases 1–4. Conversely, for the flax/epoxy composites (shown in Figure 6), there are noticeable differences in the trends. Although the range of $\sigma_{n}$ remains similar, there is a significant decrease in the $S_{A}^{+}/S_{A}$ ratio for higher $\left|\tau_{nt}\right|/{\hat{\sigma }}_{23}$ compared to the baseline. Specifically, Figure 6(a2,b2,c2) illustrates that the mean surface percentage of the interface with $\left|\tau_{nt}\right|/{\hat{\sigma }}_{23}>0.5$ is 6.95% in the baseline case (MCS case 5). This percentage decreases to 0.45% for MCS case 6 (fibre distribution variations), to 5.35% for MCS case 7 (flax fibre elastic properties variations), and to 2.40% for MCS case 8 (flax fibre shape variations). This highlights the stronger influence of elastic property variations on the $S_{A}^{+}/S_{A}$ ratio for higher $\left|\tau_{nt}\right|/{\hat{\sigma }}_{23}$ in hybrid flax/E-glass and non-hybrid flax composites under out-of-plane shear loading. Additionally, similar to the trends observed under transverse tension loading, adding E-glass fibre to flax composites increases the percentage of surface regions experiencing high stress, although the range of uncertainty is reduced.

Under transverse tension, interfacial debonding begins at the fibre equators, where the inter-fibre distances are minimal. This is followed by matrix plastic damage near the debonded regions, which eventually propagates into matrix cracks, linking the debonded areas [26,65]. Similarly, out-of-plane shear is initiated by interfacial debonding and governed by matrix plastic damage [66]. In both types of composite laminae, the interface regions exhibit higher interfacial normal stresses than interfacial shear stresses under both loadings. The interfacial normal stress plays a critical role in driving debonding mechanisms. By introducing stiff E-glass fibres into flax/E-glass composites, the material becomes capable of carrying greater loads. This increased load-bearing capacity enhances the damage tolerance by generating multiple matrix cracks, which dissipate energy and slow down the onset and subsequent development of interfacial debonding.

**Figure 7:**
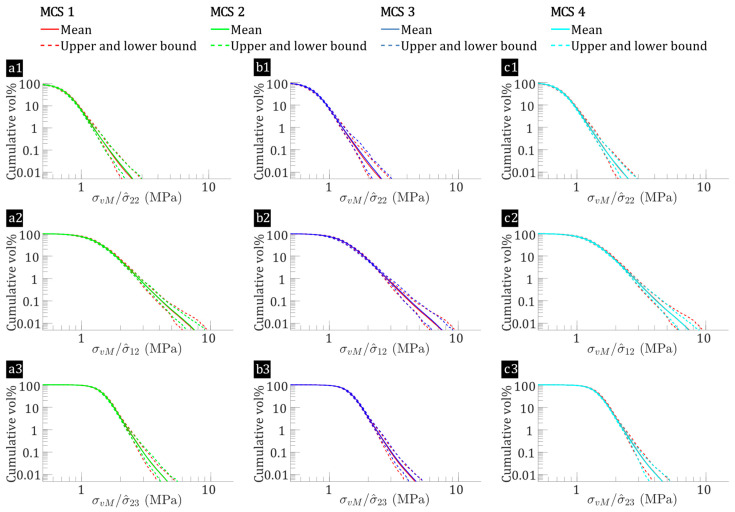
Normalised von Mises stress versus reversed cumulative matrix volume percentages for flax/E-glass composites (MCS Cases 1–4), subjected to: (**a1**,**b1**,**c1**) ${\hat{\sigma }}_{22}=1$ MPa, (**a2**,**b2**,**c2**) ${\hat{\sigma }}_{12}=1$ MPa, and (**a3**,**b3**,**c3**) ${\hat{\sigma }}_{23}=1$ MPa, where the leftmost point represents all matrix regions (100%), progressively decreasing rightward to show the proportion subjected to increasing stress, with MCS 1 (baseline) indicated by the red line, which may be partially obscured when comparing data from MCS 2, MCS 3, and MCS 4 in each subplot.

**Figure 8:**
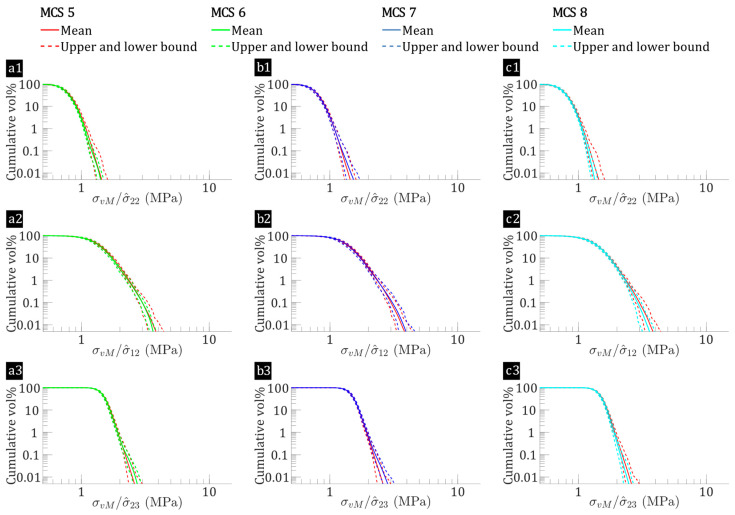
Normalised von Mises stress versus reversed cumulative matrix volume percentages for flax composites (MCS cases 5–8), subjected to: (**a1**,**b1**,**c1**) ${\hat{\sigma }}_{22}=1$ MPa, (**a2**,**b2**,**c2**) ${\hat{\sigma }}_{12}=1$ MPa, and (**a3**,**b3**,**c3**) ${\hat{\sigma }}_{23}=1$ MPa, where the leftmost point represents all matrix regions (100%), progressively decreasing rightward to show the proportion subjected to increasing stress, with MCS 5 (baseline) indicated by the red line, which may be partially obscured when comparing data from MCS 6, MCS 7, and MCS 8 in each subplot.

The effect of fibre-level variations on micro-stress fields is analysed for hybrid flax/E-glass composites and non-hybrid flax composites. Figure 7 and Figure 8 present plots of reversed cumulative matrix volume percentages against normalised von Mises stress ($\sigma_{vM}$) for flax/E-glass and flax composites, respectively. This analysis considers the transverse tensile (${\hat{\sigma }}_{22}$), in-plane shear (${\hat{\sigma }}_{12}$) and out-of-plane shear (${\hat{\sigma }}_{23}$) loadings for all 65 microstructures in each MCS case. These figures not only provide insight into the average $\sigma_{vM}$ but also outline their upper and lower bounds across all 65 microstructures. Figure 7(a1,b1,c1) highlights that flax/E-glass composites, when subjected to transverse tension, demonstrate a baseline average matrix volume percentage $\sigma_{vM}>1$ of 6.89% (bounds: 5.89% to 7.68%) in MCS case 1. This decreases to approximately 6.32% (bounds: 5.67% to 7.14%) with only fibre distribution variation (MCS case 2), representing a 7% relative decrease. While the absolute difference (0.67%) appears small, it is within the expected variability range for fibre-reinforced composites, where the fibre distribution causes minor fluctuations in stress distribution. However, the relative decrease suggests that the fibre distribution variation has a noticeable, albeit not dominant, effect on the stress concentration within the matrix. Similarly, for in-plane shear, Figure 7(a2,b2,c2) shows that the baseline (MCS case 1) has a mean volume percentage of matrix $\sigma_{vM}>2$ of about 6.66% (bounds: 6.03% to 7.45%). This is higher compared to the other cases: 6.27% (bounds: 5.45% to 6.76%) for fibre distribution variation (MCS case 2), 6.57% (bounds: 5.80% to 7.17%) for flax fibre elastic properties variation (MCS case 3), and 6.28% (bounds: 5.87% to 6.68%) for flax fibre shape variation (MCS case 4), reflecting a 6% average relative decrease.

Similarly, for flax composite laminae, as shown in Figure 8(a1,b1,c1), the baseline (MCS case 5) shows a mean matrix volume percentage with $\sigma_{vM}>1$ at approximately 3.52%, with bounds ranging from 2.46% to 4.74%. When only the fibre distribution variation is considered (MCS case 6), this mean volume percentage significantly drops to about 2.60%, with bounds from 1.92% to 3.36%, representing a 30% relative decrease. For in-plane shear, Figure 8(a2,b2,c2) shows that the baseline (MCS case 5) has a mean volume percentage of matrix with $\sigma_{vM}>2$ of around 3.86% with bounds ranging from 2.90% to 4.75%. This value is higher than those for other cases: 3.15% (bounds: 2.21% to 3.75%) for fibre distribution variation (MCS case 6), 3.37% (bounds: 2.44% to 4.33%) for flax fibre shape variation (MCS case 7), and 3.58% (bounds: 2.98% to 4.24%) for flax fibre elastic properties variation (MCS case 8), reflecting an average relative decrease of 15%. These findings suggest that fibre-level variabilities increase the percentage of highly stressed matrix regions in both hybrid flax/E-glass and non-hybrid flax composites. The effect is more pronounced in flax/epoxy laminae, with fibre distribution being the most significant factor, followed by the elastic properties and shape of the flax fibres.

When comparing flax/E-glass composites with flax composites, the former exhibits higher matrix volume percentages with increased $\sigma_{vM}$. The increased high-stress matrix regions in the flax/E-glass laminae result from intra-laminar hybridisation affecting $\sigma_{vM}$ within the matrix, as shown in Figure 7 and Figure 8. This behaviour arises from the superior stiffness of E-glass fibres relative to flax fibres, which intensifies stress concentrations, particularly under shear loading. Consequently, the inclusion of high-modulus E-glass fibres alters the matrix stress distribution and modifies the interfacial normal and shear stresses in the hybrid composites.

The damage mechanisms in the laminar structure are strongly influenced by highly localised stress regions. For a sufficiently large RVE, fibre-level variabilities show negligible influence on the RVE response at lower stress levels. However, in flax/E-glass/epoxy composites, these variabilities significantly affect the stress distribution in high-stress regions, particularly at higher stress levels. As a result, the analysis is centred on determining the maximum von Mises matrix stress ($\sigma_{vM}^{max}$), which is critical for assessing matrix failure [54].

**Figure 9:**
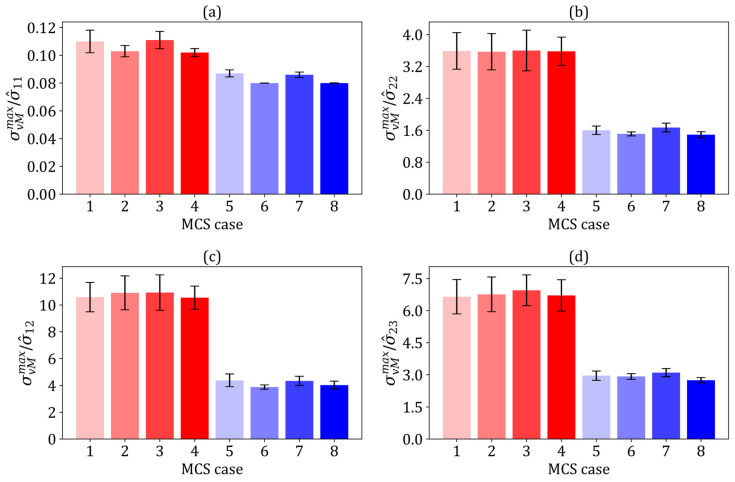
Average normalised $\sigma_{vM}^{max}$ calculated across eight Monte Carlo simulations under varying loading conditions, with colour-coded bars transitioning from light to dark red for MCS cases 1–4 (flax/E-glass composites) and light to dark blue for MCS cases 5–8 (flax composites): (**a**) ${\hat{\sigma }}_{11}=1\;MPa$, (**b**) ${\hat{\sigma }}_{22}=1\;MPa$, (**c**) ${\hat{\sigma }}_{12}=1\;MPa$ and (**d**) ${\hat{\sigma }}_{23}=1\;MPa$.

The robustness of the MCS-based stochastic model was confirmed by evaluating the convergence of the mean values (μ) and coefficient of variation (COV) for $\sigma_{vM}^{max}$ of composites in each MCS case. After 50 simulations, the averages and COVs of $\sigma_{vM}^{max}$ stabilised with minimal fluctuation, with a relative difference of less than 5%. Therefore, the selected 65 simulations for the MCS case provided sufficient accuracy for determining $\sigma_{vM}^{max}$ within the matrix. Figure 9 summarises the average $\sigma_{vM}^{max}$ under different variabilities and loading conditions. These were determined by identifying the peak von Mises stress at the integration points across all the matrix elements in each of the 65 simulations for every MCS case. The average $\sigma_{vM}^{max}$ was then computed by averaging the $\sigma_{vM}^{max}$ from all 65 simulations. The analysis of Figure 9a reveals that applying longitudinal tensile loading (${\hat{\sigma }}_{11}=1\;MPa$) results in a lower $\sigma_{vM}^{max}$, as the fibres bear the applied load along the longitudinal axis. Conversely, the in-plane shear stress (${\hat{\sigma }}_{12}=1\;MPa$) leads to a higher $\sigma_{vM}^{max}$, significantly exceeding the values observed under the out-of-plane shear stress (${\hat{\sigma }}_{23}=1\;MPa$) and transverse tensile stress (${\hat{\sigma }}_{22}=1\;MPa$).

The findings in Figure 9 reveal that flax composites (MCS cases 5–8) consistently exhibit lower average $\sigma_{vM}^{max}$ compared to flax/E-glass laminae (MCS cases 1–4). However, incorporating 12% E-glass fibres into flax composites significantly increases $\sigma_{vM}^{max}$. Specifically, Figure 9 shows that the average increases are 28% in longitudinal tensile, 129% in transverse tensile, 130% in in-plane shear, and 159% in out-of-plane shear loadings. This effect arises from the higher stiffness of the E-glass fibres, which increases the stress concentrations, especially under shear loads. Additionally, the stiffer E-glass fibres alter the matrix stress fields, influencing the interfacial normal and shear stresses and potentially affecting the intra-laminar damage mechanisms.

As shown in Figure 9, the $\sigma_{vM}^{max}$ is influenced by fibre-level variabilities, whereas the homogenised elastic constants of the composite laminae remain unaffected. In flax composites, fibre-level variabilities moderately increase $\sigma_{vM}^{max}$. The analysis of MCS cases 6 and 8 shows that variations in flax fibre shape can cause up to a 6% relative increase in the mean $\sigma_{vM}^{max}$ of flax composites. Conversely, the variability in flax fibres’ elastic properties causes a consistent increase in the mean $\sigma_{vM}^{max}$ of flax/epoxy laminae across all loading cases, reaching a 12% under in-plane shear loading. By comparison, Figure 9 shows that the effect of these fibre-level variabilities on the mean $\sigma_{vM}^{max}$ in the flax/E-glass composites (MCS cases 1–4) is minimal. In MCS case 6, where only fibre distribution variability is considered, the average relative differences in $\sigma_{vM}^{max}$ across all loading conditions are 2.8% for flax fibre elastic property uncertainties, 1.4% for cross-sectional shape uncertainties, and 2.8% for both combined.

Hybridising small-diameter E-glass fibres introduces greater uncertainty in $\sigma_{vM}^{max}$, particularly in the transverse tensile and shear loadings. As shown in Figure 9, COVs increase to 10–14% for these matrix-dominant loads, which is significantly higher than the 3–7% observed in flax composites. Fibre-level variabilities strongly influence the stress distribution, with randomness in the microstructures being the primary source of these uncertainties. This leads to COVs exceeding 10% for hybrid composite laminae (MCS case 2) and around 4% for the flax/epoxy composite laminae (MCS case 6) in $\sigma_{vM}^{max}$. This inherent variability arises from the random positioning of fibres, particularly the fixed minimum inter-fibre spacing.

The statistical analysis of the stress concentration locations within the RVEs indicates that the stress concentration does not consistently occur in regions with the smallest inter-fibre gaps. In flax composites with both fibre-level variabilities, stress concentration was observed in 12% of 65 simulations between fibres with the minimum inter-fibre spacing. The percentage increased slightly to around 15% when focusing on variabilities in flax fibre elastic properties, flax fibre shape, or excluding fibre-level variabilities. In comparison, flax/E-glass composites exhibited stress concentration in 43% of the simulations in similar regions, suggesting that incorporating E-glass fibres alters the stress distribution. Without fibre-level variabilities, considering only the variability in flax fibre elastic properties or the variability in flax fibre shape resulted in percentages of 22%, 18% and 54%, respectively. These findings highlight the crucial role of fibre-level variability in determining the stress concentration within composite laminae.

**Figure 10:**
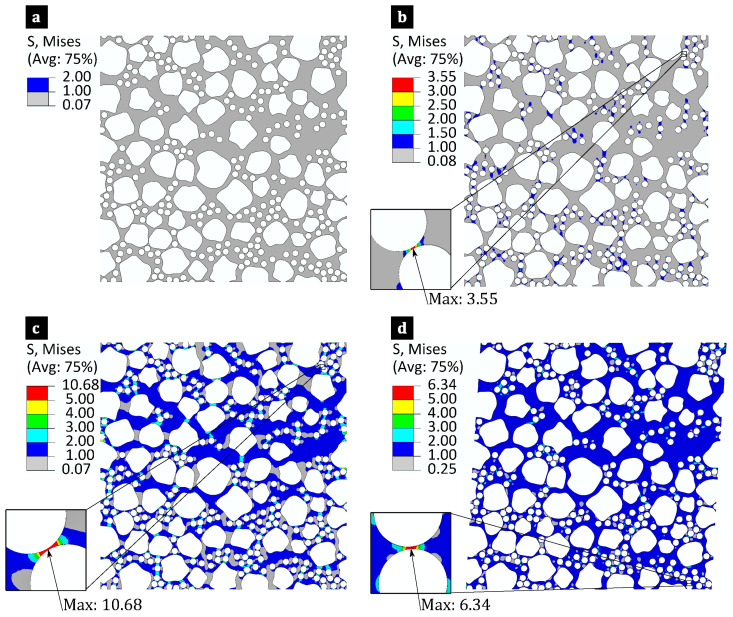
Distribution of von Mises stress within the matrix of the flax/E-glass composite (excluding fibres) with a RVE size of 580×580 μm, incorporating both fibre-level variabilities, shown for four distinct loading conditions: (**a**) ${\hat{\sigma }}_{11}=1$ MPa, (**b**) ${\hat{\sigma }}_{22}=1$ MPa, (**c**) ${\hat{\sigma }}_{12}=1$ MPa, and (**d**) ${\hat{\sigma }}_{23}=1$ MPa.

**Figure 11:**
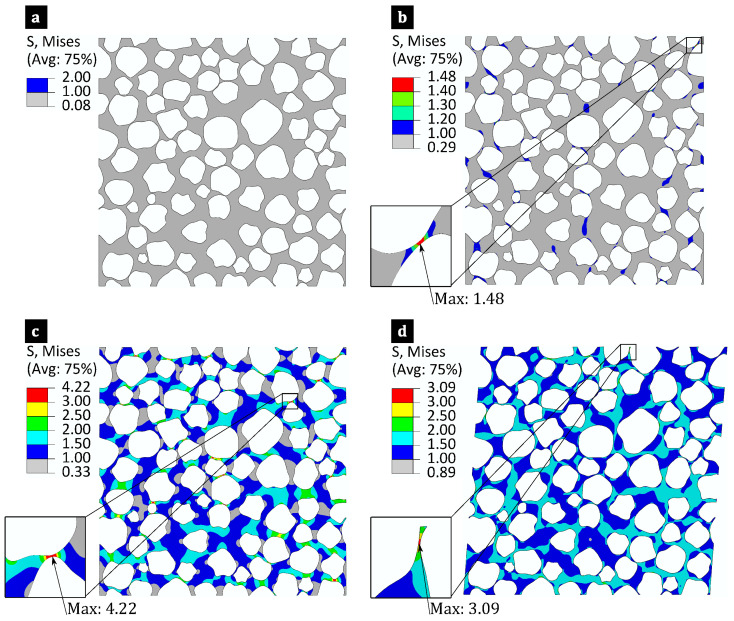
Distribution of von Mises stress fields in the flax composite (excluding fibres) with a RVE size of 580×580 μm, incorporating both fibre-level variabilities, shown for four distinct loading conditions: (**a**) ${\hat{\sigma }}_{11}=1$ MPa, (**b**) ${\hat{\sigma }}_{22}=1$ MPa, (**c**) ${\hat{\sigma }}_{12}=1$ MPa, and (**d**) ${\hat{\sigma }}_{23}=1$ MPa.

Figure 10 and Figure 11 show the von Mises stress fields within the matrix (excluding fibres) for hybrid flax/E-glass and non-hybrid flax composites under loading cases with unit macro stress components (${\hat{\sigma }}_{ij}=1\;MPa$) for baselines MCS cases 1 and 5, respectively. These figures illustrate the influence of fibre-level variability on intra-laminar stresses within the matrix. The $\sigma_{vM}^{max}$ is marked along with its locations and values for different loading cases, particularly in regions where narrow gaps exist between neighbouring fibres and the angles relative to the loading direction are minimal. This finding reinforces the role of microstructures in introducing variability in $\sigma_{vM}^{max}$.

The analysis extends to investigate the effect of fibre-level variabilities on the location of $\sigma_{vM}^{max}$ in the composites. In flax composites subjected to longitudinal tensile loading with varied flax fibre elastic properties (MCS case 7), 65 simulations reveal the following distribution for the location of $\sigma_{vM}^{max}$: 54% occur between one strong and one weak fibre, 6% between two weak fibres, and 40% between two strong fibres. Under transverse tensile loading, the respective percentages are 40%, 6%, and 54%. For longitudinal shear loading, the percentages are 6%, 38%, and 56%, while out-of-plane shear loading results in percentages of 6%, 46%, and 48%, respectively. In flax composites with varied flax fibre elastic properties and shapes (MCS case 5), a consistent trend was observed. On average, 8% of the simulations revealed the location of $\sigma_{vM}^{max}$ occurred between weak flax fibres, 44% between one strong and one weak flax fibre, and 48% between two strong flax fibres. This suggests that the location of $\sigma_{vM}^{max}$ is influenced by the flax fibre’s elastic properties, with minimal sensitivity to its shape.

In contrast, regardless of any fibre-level variabilities, the $\sigma_{vM}^{max}$ always occurs between two E-glass fibres within the matrix of flax/E-glass hybrid composites. This phenomenon arises because E-glass fibres possess higher stiffness and smaller diameters than flax fibres, leading to an uneven matrix stress distribution under load. Consequently, a higher $\sigma_{vM}^{max}$ occurs in regions between E-glass fibres due to their relative stiffness compared to flax fibres. Moreover, the low E-glass fibre volume fraction, coupled with the coexistence of flax fibres, introduce a more randomised distribution of E-glass fibres. This increased randomness may contribute to higher stress uncertainty within the matrix, unlike the lower variability observed in flax/epoxy composites.

## 4. Conclusions

The Monte Carlo simulation (MCS) stochastic homogenisation approach was applied in this study to investigate the influence of the cross-sectional shape and elastic properties of flax fibres on the homogenised elastic constants of unidirectional hybrid flax/E-glass and non-hybrid flax composite laminae. It also examined how these factors affect the distribution of interfacial stresses and von Mises matrix stress under different loading cases. Fibre-level variabilities were addressed by varying the flax fibre shape and elastic properties, which were modelled with a 20% coefficient of variation (COV) under a normal distribution, and by introducing random fibre placement within the matrix. To represent the microstructure of composite lamina with irregular flax fibres, a Non-Circular Fibre Distribution (NCFD) algorithm was developed for the automatic generation of such microstructures. This involved randomly placing non-overlapping circular E-glass fibres and irregular flax fibres within a square RVE domain.

The numerical studies revealed several key findings: (a) fibre-level variabilities—such as variations in microstructure, flax fibre shape, and flax fibre elastic properties—have little effect on the elastic properties of hybrid flax/E-glass and non-hybrid flax composites; (b) these variabilities increase the proportion of highly stressed interface regions in both composite types, with the elastic properties of flax fibre playing the most significant role; (c) the variabilities also increase the proportion of highly stressed matrix regions, particularly in flax/epoxy laminae, where fibre distribution is the most influential factor, followed by the elastic properties and shape of flax fibre; (d) introducing E-glass fibres into flax/epoxy laminae not only increases the percentage of highly stressed interface and matrix regions but also reduces the range of uncertainty and raises the uncertainty in maximum von Mises matrix stress; (f) for flax composites, the elastic properties of the flax fibres have a dominant effect on the maximum von Mises matrix stress, with geometric variability playing a minor role.

Overall, the findings demonstrate that for the purpose of determining homogenised elastic constants ($E_{ijkl}$, $G_{ijkl}$, $\nu_{ijkl}$) of natural/synthetic fibre reinforced hybrid composites (NFHCs), the shape and elastic properties of the natural fibre can be considered negligible. In future work, experimental data for the hybrid flax/E-glass composite could be used to validate the assumptions and simplifications used in the RVE model and Monte Carlo analysis presented in this study. However, accounting for fibre-level variabilities in elastic properties, shape, and especially microstructure, is crucial for ensuring reliable stress analysis, which determines the damage evolution and failure stress of the hybrid fibre composite. Additionally, intra-laminar hybridisation tends to increase the number of highly stressed regions, necessitating careful consideration when working with such heterogeneous composite systems.

## Figures and Tables

**Table 1:** Tensile modulus, diameter and density of flax fibres [27,45,46,47,48,49].

Tensile Modulus $\left(GPa\right)$	Diameter, d (μm)	Density, ρ$\;({g/cm}^{3})$	Reference
50–90	10–50	1.4–1.5	[27]
60–80	5–38	1.4	[45]
27.5–85	24	1.5	[46]
30.1	160–185	1.4	[47]
50–70	50–100	1.5	[48]
27.6	25	1.5	[49]

**Table 2:** Material properties of constituents [27,55,57,58]: mean values, standard deviations, coefficient of variation, and corresponding distribution.

	μ	σ	COV (%)	Distribution Type
*Epoxy*				
E (GPa)	2.55	−	−	Constant
ν	0.35	−	−	Constant
*E-glass fibre*				
E (GPa)	73	−	−	Constant
ν	0.2	−	−	Constant
d (μm)	15	−	−	Constant
*Flax fibre*				
$E_{1}$ (GPa)	52	10.4	20%	Normal
$E_{2}$ (GPa)	7	1.4	20%	Normal
$G_{12}$ (GPa)	3	0.6	20%	Normal
$G_{23}\;$(GPa)	2	0.4	20%	Normal
$\nu_{12}$	0.3	0.06	20%	Normal
$\nu_{23}$	0.75	0.15	20%	Normal
d (μm)	58	11.6	20%	Normal

**Table 3:** Monte Carlo uncertainty analysis: case parameters.

Case	$\left(V_{fF},{V_{fE},V}_{m}\right)$	Number of Simulations	Cross-Sectional Shape of Flax Fibre	Flax Fibre Shape Variability	Flax Fibre Elastic Properties Variability
1	(0.48, 0.12, 0.4)	65	Irregular	±20%	±20%
2	(0.48, 0.12, 0.4)	65	Circular	No	No
3	(0.48, 0.12, 0.4)	65	Circular	No	±20%
4	(0.48, 0.12, 0.4)	65	Irregular	±20%	No
5	(0.60, 0, 0.4)	65	Irregular	±20%	±20%
6	(0.60, 0, 0.4)	65	Circular	No	No
7	(0.60, 0, 0.4)	65	Circular	No	±20%
8	(0.60, 0, 0.4)	65	Irregular	±20%	No

**Table 4:** Comparative analysis of mesoscopic elastic constants of flax and E-glass composites derived from RVE (sample size: 5), Halpin-Tsai predictions [62] and experimental data [60,61].

Composite	Method	${\hat{E}}_{11}$ (GPa)	${\hat{\nu }}_{12}$	${\hat{E}}_{22}$ (GPa)	${\hat{G}}_{12}$ (GPa)	${\hat{G}}_{23}$ (GPa)
Flax/epoxy	RVE (μ ±3σ)	31.68±0.01	0.32±0.01	3.70±0.01	1.46±0.01	1.16±0.01
	Halpin-Tsai	31.84	0.32	3.30	1.39	1.38
	Experimental	34.40±2.60	N/A	N/A	N/A	N/A
E-glass/epoxy	RVE (μ ±3σ)	45.56±0.01	0.24±0.01	13.86±0.20	5.62±0.12	4.88±0.09
	Halpin-Tsai	45.74	0.26	15.12	4.62	4.62
	Experimental	45.60	0.278	16.20	5.83	5.79

## Data Availability

Dataset available on request from the authors.
